# A Fibre-Reinforced Poroviscoelastic Model Accurately Describes the Biomechanical Behaviour of the Rat Achilles Tendon

**DOI:** 10.1371/journal.pone.0126869

**Published:** 2015-06-01

**Authors:** Hanifeh Khayyeri, Anna Gustafsson, Ashley Heuijerjans, Marko K. Matikainen, Petro Julkunen, Pernilla Eliasson, Per Aspenberg, Hanna Isaksson

**Affiliations:** 1 Department of Biomedical Engineering, Lund University, Lund, Sweden; 2 Department of Biomedical Engineering, Eindhoven University of Technology, Eindhoven, Netherlands; 3 Department of Mechanical Engineering, Lappeenranta University of Technology, Lappeenranta, Finland; 4 Department of Clinical Neurophysiology, Kuopio University Hospital, Kuopio, Finland; 5 Department of Applied Physics, University of Eastern Finland, Kuopio, Finland; 6 Department of Clinical and Experimental Medicine, Linköping University, Linköping, Sweden; National University of Ireland Galway, IRELAND

## Abstract

**Background:**

Computational models of Achilles tendons can help understanding how healthy tendons are affected by repetitive loading and how the different tissue constituents contribute to the tendon’s biomechanical response. However, available models of Achilles tendon are limited in their description of the hierarchical multi-structural composition of the tissue. This study hypothesised that a poroviscoelastic fibre-reinforced model, previously successful in capturing cartilage biomechanical behaviour, can depict the biomechanical behaviour of the rat Achilles tendon found experimentally.

**Materials and Methods:**

We developed a new material model of the Achilles tendon, which considers the tendon’s main constituents namely: water, proteoglycan matrix and collagen fibres. A hyperelastic formulation of the proteoglycan matrix enabled computations of large deformations of the tendon, and collagen fibres were modelled as viscoelastic. Specimen-specific finite element models were created of 9 rat Achilles tendons from an animal experiment and simulations were carried out following a repetitive tensile loading protocol. The material model parameters were calibrated against data from the rats by minimising the root mean squared error (RMS) between experimental force data and model output.

**Results and Conclusions:**

All specimen models were successfully fitted to experimental data with high accuracy (RMS 0.42-1.02). Additional simulations predicted more compliant and soft tendon behaviour at reduced strain-rates compared to higher strain-rates that produce a stiff and brittle tendon response. Stress-relaxation simulations exhibited strain-dependent stress-relaxation behaviour where larger strains produced slower relaxation rates compared to smaller strain levels. Our simulations showed that the collagen fibres in the Achilles tendon are the main load-bearing component during tensile loading, where the orientation of the collagen fibres plays an important role for the tendon’s viscoelastic response. In conclusion, this model can capture the repetitive loading and unloading behaviour of intact and healthy Achilles tendons, which is a critical first step towards understanding tendon homeostasis and function as this biomechanical response changes in diseased tendons.

## Introduction

The Achilles tendon is the largest tendon in the body and the most commonly injured tendon [1]. It is vulnerable to injury due to the high forces it has to withstand and its limited vascularity. Chronic tendon injuries, *tendinopathies*, are thought to be a form of mechanically induced degradation of the tissue matrix that gives rise to pain and can lead to ruptures [2, 3]. Yet, the best treatments of tendinopathies and ruptures remain unresolved. This is partly due to our limited knowledge in the basic structural and compositional properties of Achilles tendons [4] and how these control the tendon biomechanical behaviour.

Tendons are composed of principally 70% water and 30% collagens [5], where collagen I constitutes 90% of the dry weight of Achilles tendons. Collagen molecules have been shown to confer the mechanical tensile strength observed in experiments of tendons [6]. The collagens have a complex hierarchical structure where the collagen fibrils are packed in parallel bundles along the axis of the tendon to create collagen fibres, which in turn are parcelled in fascicles and fibre bundles creating a multi-hierarchical tissue structure. The rest of the dry tissue matrix (non-fibrillar matrix) consists of a small amount of elastin (~2%) [4] and proteoglycans (~1%) [7]. Despite the small amount of non-fibrillar extracellular component any disruption of the tendon constituents (water content, collagen and non-fibrillar matrix) can be detrimental to tissue function and lead to ruptures. Therefore, understanding the relationship between structure, composition and function in healthy Achilles tendons is essential as it can act as a benchmark when testing novel treatment strategies. In this regard, biomechanical computer models can help explain the complex biology of Achilles tendon structure and composition and the synergies for functional load-bearing.

Existing biomechanical models of tendons are often generalised and treat tendon and ligament behaviour concurrently as soft musculoskeletal collagenous tissues. However, studies have shown that although the tissues are similar in their structure and composition, their extracellular matrix that provides the mechanical response is function-dependent [8]. Moreover, tendons with more energy storing capacity are better at resisting damage compared to those with lower energy storing function, which illustrates that tendon biomechanical behaviour is dictated by its function at its anatomical location [9]. Biomechanical tendon models are often divided into either a macroscopic or microscopic level behaviour of the tissue. For example, the macroscopic mechanical behaviour has been described in constitutive models by characterising e.g. hyperelasticity, viscoelasticity and poroelasticity of tendons [10–14]. On the microscopic level, the focus has been on the collagen fibrils [15, 16] and their wavy, spiral pattern referred to as crimp [17, 18]. During tension of the tendon, the crimped collagen fibres are initially stretched out, and additional tension can lead to microscopic damage and failure of the collagen fibrils [19]. The proposed continuum models are based on constitutive laws and appropriate strain-energy functions to capture the tendon biomechanical behaviour. However, experiments show a large structure-composition variability in tendons which continuum models cannot entail in the classic strain-energy function [20]. Moreover, tendon injuries are correlated with collagen function and homeostasis. Therefore, structural models that distinguish between the tissue matrix and collagen fibres are essential.

The importance of multi-structural models of tendons and soft tissues was first demonstrated by Lanir [21] and most recently by Fan and Sacks [22]. They described the biomechanical behaviour of the tendons by modelling the microscopic constituents and the mechanical interactions between the tissue components, such as tissue matrix and fibre orientation, where the responses add up to the whole tissue macroscopic behaviour. Other researchers have introduced structural strain-energy functions which account for the structural changes in the tissue, also on the micro scale [23, 24]. By doing so they have been able to represent collagen fibre biomechanics and fibre-recruitment based on loading history. Further, Calvo, Pena [25] described tissue damage in a continuum model where damage was based on separate contributions from both the fibre and matrix component. Rodriguez, Cacho [26] on the other hand adopted statistical distributions based on the extension of the fibre bundles to depict fibre damage. But neither of the existing models considered the biphasic behaviour of the tissue, where the viscoelastic behaviour of tendons is partly due to the movement of water within the tissue [1]. Lavagnino, Arnoczky [27] developed a poroelastic model consisting of a global tissue model and a local model of a tenocyte surrounded by extracellular matrix. This model was able to predict the stresses and fluid exudation from the tendon as well as correlate the simulated local cell strains to increased collagenase gene expression observed experimentally. Biphasic models can more accurately consider the local stresses on the extracellular matrix that is necessary when studying cell mechanobiology, and damage and failure criteria [28]. Thus, it is reasonable to develop a structural poroviscoelastic model for tendons that also accounts for the fluid phase. This type of model has been developed for articular cartilage, another tissue rich in collagen [29–31] where the authors were able to successfully predict the effects of collagen fibre morphology on cartilage damage and depth-dependent behaviour.

This study adapts the poroviscoelastic model developed by Wilson, van Donkelaar [31] for articular cartilage to describe the biomechanical behaviour of the rat Achilles tendon. The material model divides the Achilles tendon into three main constituents, namely fluid, collagen fibre and non-fibrillar matrix. We investigate if this model can capture the biomechanical behaviour of rat Achilles tendons as observed in cyclic tensile loading experiments, thereby predicting the mechanical role of the different tissue constituents under loading. The model will be tested with experimental data from the study by Eliasson, Fahlgren [32]. In their study, the Achilles tendons of 9 Sprague-Dawley rats were harvested at 16 weeks of age (control group in the published study) and subsequently subjected to mechanical testing. The tendons were subjected to cyclic tensile loading between 1–20 N with a rate of 0.1 mm/s. This cycle was repeated 20 times before a final failure test was performed, see original publication for more details.

The results demonstrate that our novel multi-structural material model for the rat Achilles tendon has the capacity to consider the role that collagen fibril morphology and collagen viscoelasticity play in the overall biomechanical behaviour of the tissue. The study shows how different tissue constituents play a part in resisting tensile loading and predicts the relaxation and recovery response of rat Achilles tendons.

## Materials and Methods

### Material model for the Achilles tendon

A constitutive model for the Achilles tendon was developed based on an existing material model for articular cartilage [29, 31]. The tendon is described as a biphasic tissue in which the solid matrix is divided into a non-fibrillar part, describing the proteoglycan matrix, and one fibrillar part depicting the collagen fibres. Assuming that the porous solid matrix is fully saturated with water, the total stress in the tendon tissue is given by

$$\sigma_{total}=\sigma_{s}-pI=\sigma_{f}+\sigma_{m}-pI,$$ (1)

where ***σ***_*s*_ is the stress in the solid matrix, *p* is the hydrostatic pressure, **I** the unit tensor. In turn, ***σ***_*f*_ and ***σ***_*m*_ are the stresses in the collagen fibres and the non-fibrillar matrix respectively.

**Fig 1:**
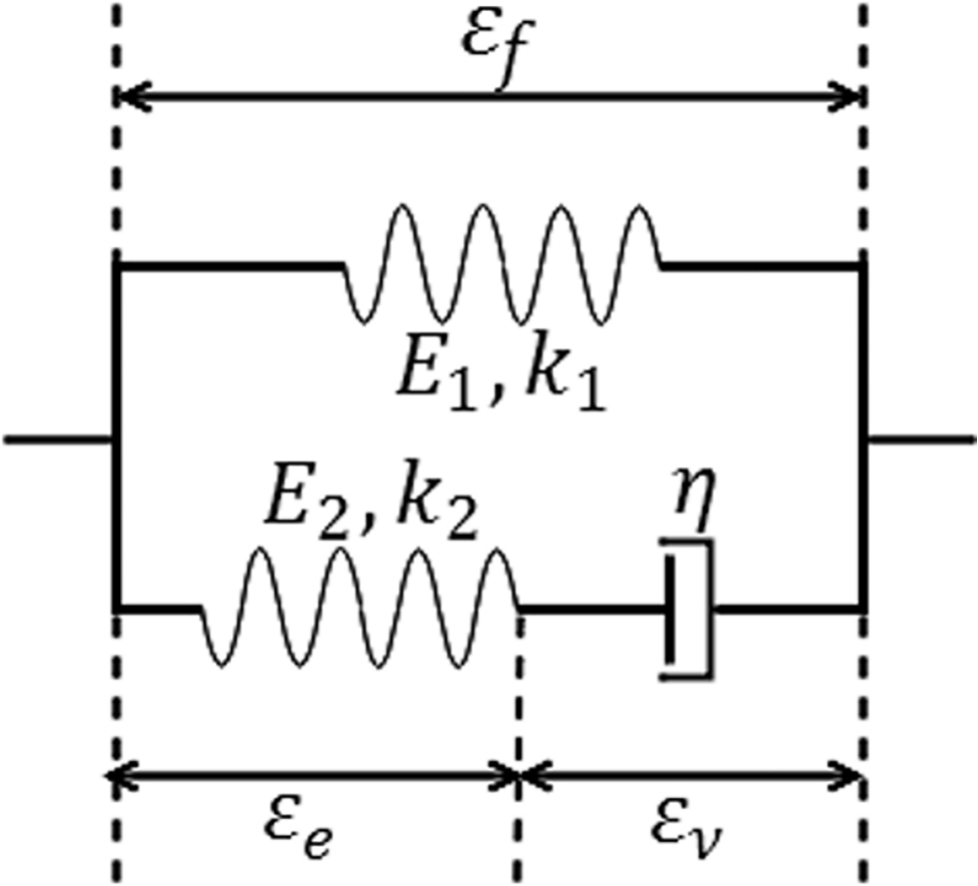
An illustration of the viscoelastic model representing the collagen fibres. *E*_1_, *E*_2_, *k*_1_, *k*_2_ and *η* are system constants, *ε*_*f*_ stands for the total fibre strain, *ε*_*v*_ is the strain in the dash pot and *ε*_*e*_ is the strain in the spring in the Maxwell element.

Collagen fibres exhibit a viscoelastic response during loading [33–35]. To capture this behaviour, a modified standard linear solid model was used containing one spring connected in parallel with a Maxwell element (Fig 1). An exponential stress-strain relationship, as introduced by Wilson, van Donkelaar [30], was used for both springs in the fibre model.

Assuming that the fibres only carry load in tension, the stress in the single spring system (top spring in Fig 1) is given by

$$P_{1}=E_{1}\left(e^{k_{1}\varepsilon_{f}}-1\right)\;\varepsilon_{f}>0$$ (2)

and the stress in the Maxwell element is

$$P_{2}=E_{2}(e^{k_{2}\varepsilon_{e}}-1)=\eta {\dot{\varepsilon }}_{\nu }\;\varepsilon_{e}>0,$$ (3)

where *P*_1_ and *P*_2_ are the first Piola-Kirchoff stresses, *E*_1_, *E*_2_, *k*_1_ and *k*_2_ are stiffness constants and *η* is the damping constant. The total fibre strain (*ε*_*f*_) equals the sum of the strains in the dashpot (*ε*_*v*_) and in the spring (*ε*_*e*_) in the Maxwell element. Thus, the total fibre stress (*P*_*f*_) is given by

$$P_{f}=P_{1}+P_{2}=P_{1}+\eta {\dot{\varepsilon }}_{\nu }=P_{1}+\eta ({\dot{\varepsilon }}_{f}-{\dot{\varepsilon }}_{e}).$$ (4)

$$\varepsilon_{e}=\frac{1}{k_{2}}\ln\left(\frac{P_{2}}{E_{2}}+1\right),$$ (5)

$${\dot{\varepsilon }}_{e}=\frac{{\dot{P}}_{2}}{k_{2}(P_{2}+E_{2})}$$ (6)

Expressed as a function of the total fibre strain (*ε*_*f*_), the total fibres stress becomes (Eq 7)

$${P_{f}=P}_{1}+\eta ({\dot{\varepsilon }}_{f}-\frac{{\dot{P}}_{2}}{k_{2}(P_{2}+E_{2})})=P_{1}+\eta ({\dot{\varepsilon }}_{f}-\frac{\left({\dot{P}}_{f}-{\dot{P}}_{1}\right)}{k_{2}(P_{f}-P_{1}+E_{2})}).$$ (7)

After time integration with the backward Euler method, the expression above (Eq 7) takes the form of a quadratic equation, which has the following (positive) solution

$${P_{f}}^{t+∆t}=-\frac{b}{2}+\frac{1}{2}\sqrt{b^{2}-4c},$$ (8)

Where

$$b=E_{2}-2P_{1}-\eta {\dot{\varepsilon }}_{f}+\frac{\eta }{k_{2}\Delta t}$$ (9)

And

$$c=\frac{\eta }{k_{2}\Delta t}P_{f}^{t}-(P_{1}+\eta {\dot{\varepsilon }}_{f})(E_{2}-P_{1})-\frac{\eta }{k_{2}}{\dot{P}}_{1},$$ (10)

*P*_*f*_^*t*+Δ*t*^ is the fibre stress in the current time increment and *P*_*f*_^*t*^ is the fibre stress from the previous time increment.

Finally, the Cauchy stress tensor for the collagen fibres in the solid tendon matrix (***σ***_*f*_ in Eq 1) was given by

$$\sigma_{f}=\frac{\lambda }{J}P_{f}e_{f}{e_{f}}^{T},$$ (11)

where *λ* is the fibre stretch, *J* the determinant of the deformation tensor **F** and ***e***_***f***_ is a one-dimensional unit vector describing the current fibre orientation [29].

Since tendons undergo large deformations the non-fibrillar component of the solid matrix was modelled as a compressible neo-Hookean material. The energy function (*W*) suggested by Simo and Ortiz [36] and previously adopted for porous solids and biological tissues [30] was used (Eq 12).

$$W=\frac{K_{m}}{2}(\frac{1}{2}\;(J^{2}-1)-\ln\left(J\right)\;)+\frac{G_{m}}{2}(tr(C)-3det{\left(C\right)}^{1/3}),$$ (12)

where **F** is the deformation tensor and **C** is the right Cauchy-Green tensor. The bulk (*K*_*m*_) and the shear (*G*_*m*_) moduli of the matrix are defined as

$$K_{m}=\frac{E_{m}}{3(1-2\upsilon_{m})},$$ (13)

$$G_{m}=\frac{E_{m}}{2(1+\upsilon_{m})}.$$ (14)

*E*_*m*_ is the Young’s modulus and *υ*_*m*_ is Poisson’s ratio of the non-fibrillar matrix. The Cauchy stress in the non-fibrillar matrix (***σ***_***m***_) was derived as

$$\sigma_{m}=\frac{2}{J}F\frac{\partial W}{\partial C}F^{T}=\frac{K_{m}}{2}(J-\frac{1}{J})I+\frac{G_{m}}{J}(FF^{T}-J^{2/3}I).$$ (15)

The permeability (*k*) of tendons was assumed to be void ratio-dependent [37] and followed

$$k=k_{0}{\left(\frac{1+e}{1+e_{0}}\right)}^{M_{k}},$$ (16)

where *k*_0_ is the initial permeability, *M*_*k*_ a positive constant and *e* and *e*_0_ the current and initial void ratios [30, 38]. The void ratio in a porous medium is defined as the volume ratio between the fluid volume fraction (*n*_*f*_) and the solid volume fraction (*n*_*s*_ = 1 − *n*_*f*_). The total fluid volume fraction can be estimated from the water mass fraction (*n*_*f*,*m*_) [29], so that

$$n_{f}=\frac{\rho_{s}n_{f,m}}{1-n_{f,m}+n_{f,m\;}\rho_{s}},$$ (17)

where *ρ*_*s*_ is the solid tissue density, calculated from the tendon experiments (data from Eliasson, Fahlgren [32] as 1.4g/ml). This was implemented in a finite element software (Abaqus), which required only the initial void ratio. The current void ratio was computed by Abaqus’ Soils Analysis procedure, used for poroelastic materials.

### Finite element modelling of the Achilles tendons

Specimen-specific finite element models were created of 9 rat Achilles tendons using geometries measured in the experiments by Eliasson, Fahlgren [32]. The finite element models were created in Abaqus (v6.12–4, Dassault Systèmes, France) and the constitutive model for the fibre-reinforced solid matrix was implemented in an UMAT subroutine. All tendons were assumed to be cylindrical, constant cross-sectional area along the lengths and assigned with axisymmetric pore pressure elements (CAX4P).

**Fig 2:**
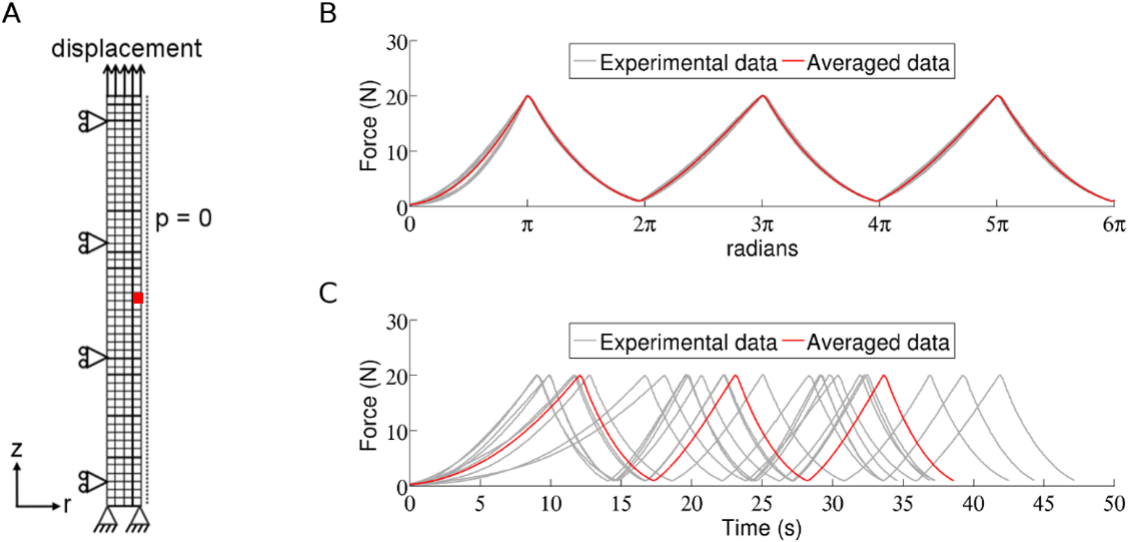
Model illustrations. A) Mesh and boundary conditions. B) The experimental loading protocol cycles 1–3 for all 9 tendons interpolated over 2π for each load cycle (grey). The average loading protocol for cycles 1–3 (red). C) The loading protocols for cycle 1–3 for all 9 tendons in the time-domain (grey) illustrate the variability among experimental specimens. The average loading protocol for cycles 1–3 in time-domain (red).

Boundary conditions were modelled following the experimental set up in the mechanical tensile tests. The finite element nodes at the base of the tendon models (calcaneus bone end) were modelled with encastre boundary conditions to represent the clamp in the mechanical testing machine (Fig 2A). In the experiments [32], during each load cycle the tendons were displaced with 0.1 mm/s from 1 N until 20 N force was obtained, and displacements and forces were recorded. This was translated to the computational tendon model by applying the recorded displacements from the experiment on the top nodes of the tendon model (gastrocnemius muscle end). The force in the tendon was computed and material parameters of the constitutive model were optimised to fit the computed force to the experimental force data (detailed description of the scheme is outlined in the next section). The nodes along the symmetry axis of the tendon finite element model were confined in the radial direction and zero pore pressure was prescribed along the free edge, allowing fluid to flow over the boundary (Fig 2A).

The experimental data exhibited large temporal variation in the tendons’ response to cyclic loading. Therefore, to create an average tensile loading protocol the tensile data from the 9 tendons were translated to the frequency domain by interpolating each load cycle over 2π (Fig 2B and 2C). An average force curve as well as displacement curve was calculated in the frequency domain, using data from all 9 tendons, before transforming the average data back to time domain by interpolating over the average time period for each load cycle. This procedure created an average tensile loading protocol for cycles 1–3 and another average protocol for cycles 10–12. Additionally, an average tendon finite element mesh was created based on the average geometrical measures from the experiments (mean length: 8.4 mm, and cross-sectional area: 1.63 mm^2^).

### Calibration of Achilles tendon models with experimental data

**Fig 3:**
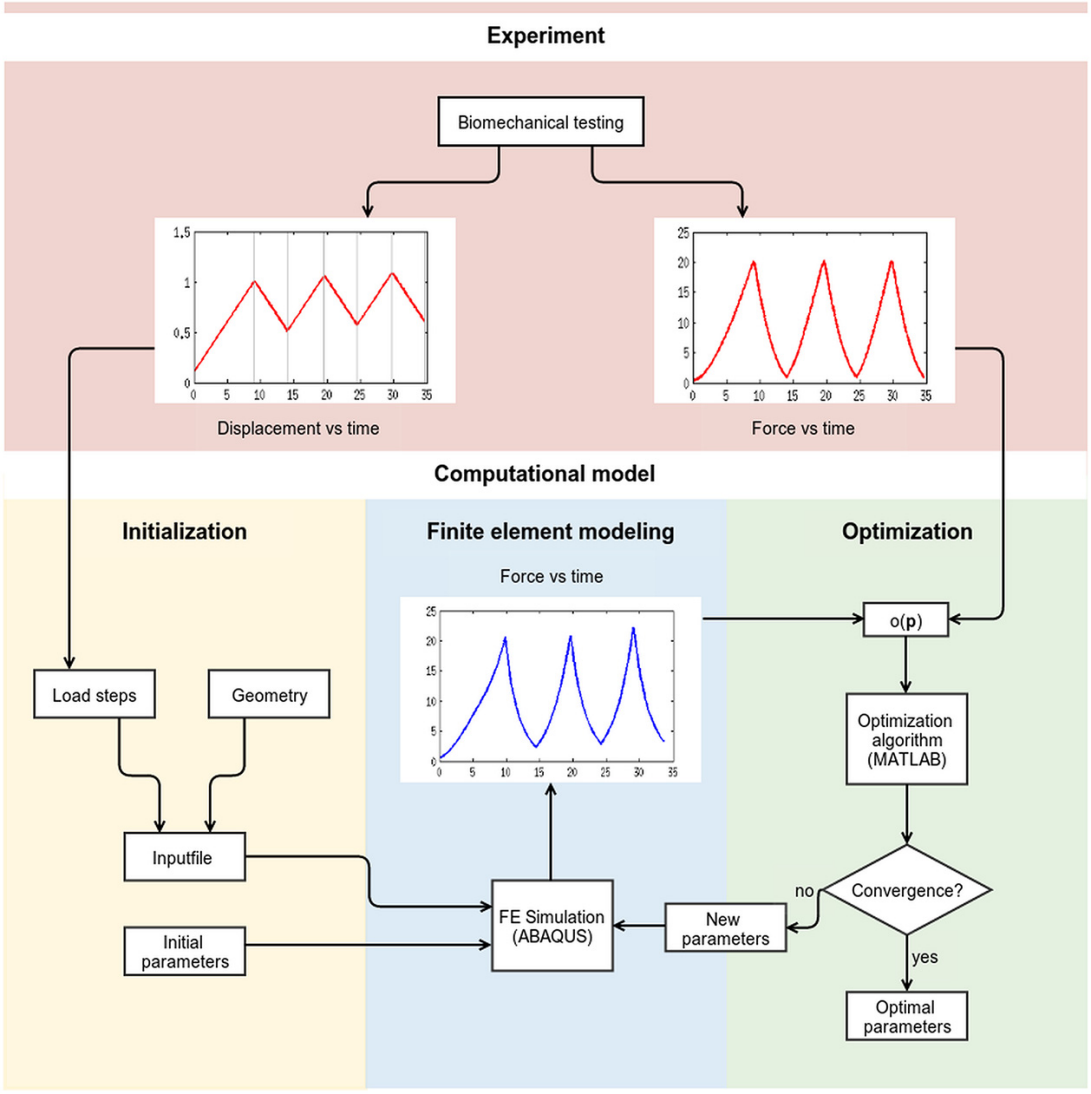
A schematic picture showing the optimisation procedure.

The proposed constitutive material model was calibrated to the data from the mechanical tensile test following an iterative computational scheme (Fig 3), which was run for each of the 9 tendons of the experiment. For each tendon, the experimental displacement protocol was applied as boundary condition on the specimen-specific FE tendon model to simulate the experiment. The reaction forces of the tendon were computed during the simulation and compared to the forces recorded during the experiment (Fig 3, Finite element modelling). If the difference between the simulated forces and those from the experiment were too large, new constitutive model parameters were proposed before a new iteration began (Fig 3, Optimisation). This optimisation scheme was run until convergence, i.e. the model calibration could not improve further and the change in the objective value function was less than 10^–5^. The tendon model was fitted to experimental data by optimising the nine unknown constitutive model parameters (*E*_1_, *E*_2_, *k*_1_, *k*_2_, *η*, *k*_0_, *M*_*k*_, *E*_*m*_, *v*_*m*_).

Three load cycles from different time points of the experiment were used to calibrate the data: (1) cycle 1–3 where large variations are seen between the cycles and the viscoelastic properties are very pronounced and (2) cycle 10–12 which assumingly correspond to preconditioned tendons. Displacement controlled loads were applied with a strain rate of 0.1 mm/s, corresponding to experimental protocol.

In the FE simulations, Abaqus’ total Lagrangian formulation and the NLGEOM key was used to account for the large deformations and the geometrical nonlinearities when implementing the material model for the fibre-reinforced solid matrix in the UMAT subroutine. In the initial configuration, all fibre direction vectors (***e*** ^0^) were directed in parallel with the tendon axis. The directional fibre vectors were updated based on the deformation gradient (**F**) in each iteration of the Abaqus solver (Eq 18).

$$e^{n+1}=F\;e^{0}.$$ (18)

The fibre vectors defined the fibres stretch, (*λ*) which was used to calculate the logarithmic strain (Eq 19).

$$\varepsilon_{f}=\ln\left(\lambda \right)=ln(\left|e^{n+1}\right|).$$ (19)

The reaction forces from the Abaqus simulations were fed into Matlab where an unconstrained nonlinear minimisation algorithm was used for optimising the unknown material parameters. This was done by minimising the objective function (*f* = *o*(***p***) in Fig 3, Eq 20) defined as the mean squared error between the model reaction force and the reaction force measured in the experiment:

$$\min\;f=\frac{1}{6}\sum_{i=1}^{6}\frac{1}{n_{i}}(\sum_{j=1}^{n_{i}}{\left({\left(F_{mod}\right)}_{j}-{\left(F_{exp}\right)}_{j}\right)}^{2}),$$ (20)

where *F*_*mod*_ is the reaction force from the finite element analysis, *F*_*exp*_ is the reaction force measured in the experiment and *n*_*i*_ is the number of data points in each load step.

### Testing the predictive capacity of the tendon model

To test the model’s ability to predict tendon biomechanical behaviour reported in literature, new FE simulations were carried out on the average tendon model using the optimised model parameters for the constitutive model. The following tests were performed:

- **Strain-stiffening test:** Tensile loads with strain rates of 0.05, 0.1, 0.2, 0.4, 0.6 and 1.0 mm/s. The displacements on the top nodes of the tendon corresponded approximately to the first load peak in the experimental data.
- **Creep test:** Tensile load at 1, 2 and 3 MPa was applied for 500 s before the load was decreased to 0.2 MPa and held constant for an additional 500 s for the tendon to recover.
- **Stress-relaxation test:** Simulations with 2, 4, 6, 8 and 10% strain were applied at 0.1 mm/s followed by a relaxation period of 300 s respectively.
- **Testing the non-fibrillar matrix:** To test the contribution of the saturated non-fibrillar matrix to the overall biomechanical response of the tendons, the collagen fibres were rearranged to run in the horizontal direction so that they could not carry any tensile loads before the stress-relaxation test protocol described above was applied on the tendon.

## Results

**Fig 4:**
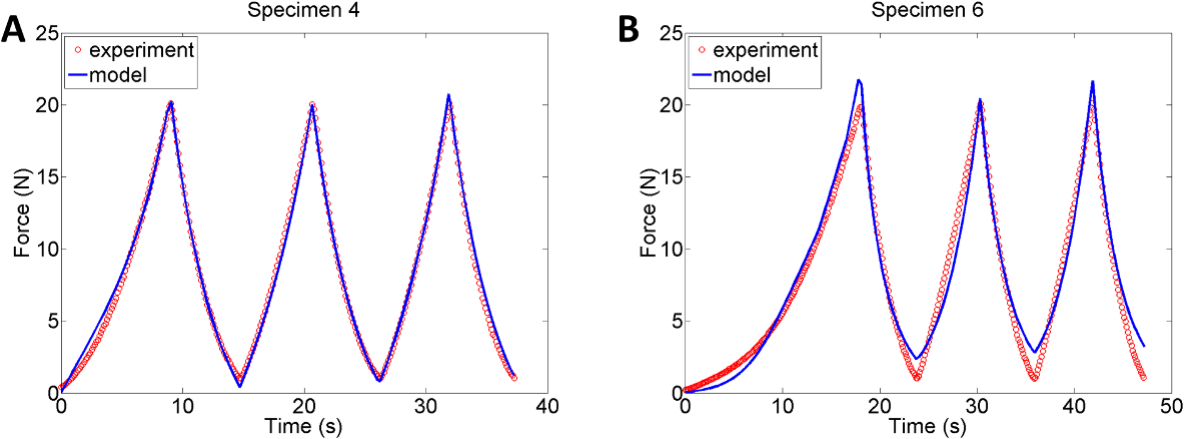
Model calibration. The new poroviscoelastic model fitted to experimental data from cycle 1–3 of the tensile tests on rat Achilles tendons. The best (A) material model fit (RMS = 0.42) and the worst (B) material model fit (RMS = 1.02).

The poroviscoelastic fibre-reinforced model developed in this study is able to capture the loading and unloading behaviour of rat Achilles tendons with good accuracy (Root Mean Square (RMS) between 0.42 and 1.02), see Fig 4. The material model was optimised to 9 specimen-specific FE-models and loading conditions, and captures the large variability in Achilles tendon biomechanical behaviour that was observed in the experiments, see Table 1.

**Table 1:** The optimised model parameters for all 9 specimen-specific finite element models based on cycle 1–3.

specimen	*RMS*	*E*_1_ (MPa)	*E*_2_ (MPa)	*k*_1_	*k*_2_	*η* (MPa∙s)	*k*_0_ (mm/s)	*M*_*k*_	*E*_*m*_ (MPa)	*v*_*m*_
**1**	0.699	0.017	0.233	38.49	29.61	582.01	3.96E-07	0.21	0.56	0.24
**2**	0.834	0.074	1.683	44.91	25.97	1103.10	5.87E-10	0.59	0.57	0.21
**3**	0.869	0.017	0.380	44.95	35.78	595.58	1.82E-07	0.67	0.70	0.40
**4**	0.421	0.648	6.251	25.85	9.14	336.59	7.65E-10	0.45	1.00	0.49
**5**	0.854	0.023	0.452	48.50	37.01	943.17	5.47E-10	0.55	0.56	0.14
**6**	1.021	0.003	0.030	36.13	38.03	537.40	1.39E-09	1.40	0.59	0.34
**7**	0.819	0.027	3.001	42.81	16.50	484.60	3.04E-08	0.001	0.70	0.44
**8**	0.920	0.001	0.054	44.54	34.45	770.67	6.22E-10	0.78	0.65	0.20
**9**	0.731	0.022	0.452	42.96	31.86	897.18	1.39E-07	0.47	0.95	0.19
**mean**	-	0.092	1.393	41.02	28.71	694.48	8.34E-08	0.57	0.70	0.29
**SD**	-	0.209	2.063	6.77	9.93	248.73	1.36E-07	0.39	0.17	0.13
**CV**	-	2.26	1.48	0.16	0.35	0.36	1.63	0.68	0.24	0.43

The mean (of the 9 optimised tendon models), the standard deviations (SD) and the coefficients of variation (CV) of the specimens are calculated.

**Fig 5:**
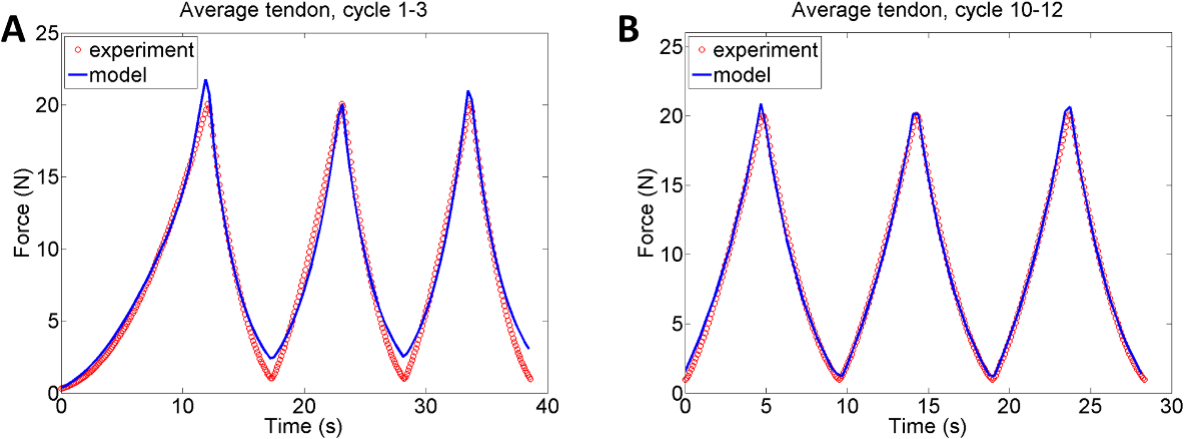
Average tendon model. The optimised result when the material model is fitted to the average tendon model and the average loading protocol. A) Loading cycle 1–3, RMS = 0.84 and B) during later loading cycles (cycle 10–12), RMS = 0.41.

The experimental data agreed well with the average tendon model and the average tensile loading protocol created in this study, see Fig 5A. Simulations of later loading cycles (cycle 10–12), which assumed that the collagen fibres are preconditioned in the specimens of the experiment, show a further improvement of the optimised biomechanical model with an even further reduced RMS (Table 2 and Fig 5B).

**Table 2:** The optimised model parameters for the average tendon model based on cycle 1–3 and 10–12.

cycle	*RMS*	*E*_1_ (MPa)	*E*_2_ (MPa)	*k*_1_	*k*_2_	*η* (MPa∙s)	*k*_0_ (mm/s)	*M*_*k*_	*E*_*m*_ (MPa)	*v*_*m*_
**1–3**	0.837	0.023	0.443	40.00	31.06	609.34	1.19E-07	0.96	0.77	0.35
**10–12**	0.413	0.024	7.009	38.00	11.63	413.23	1.75E-09	0.84	0.37	0.17

**Fig 6:**
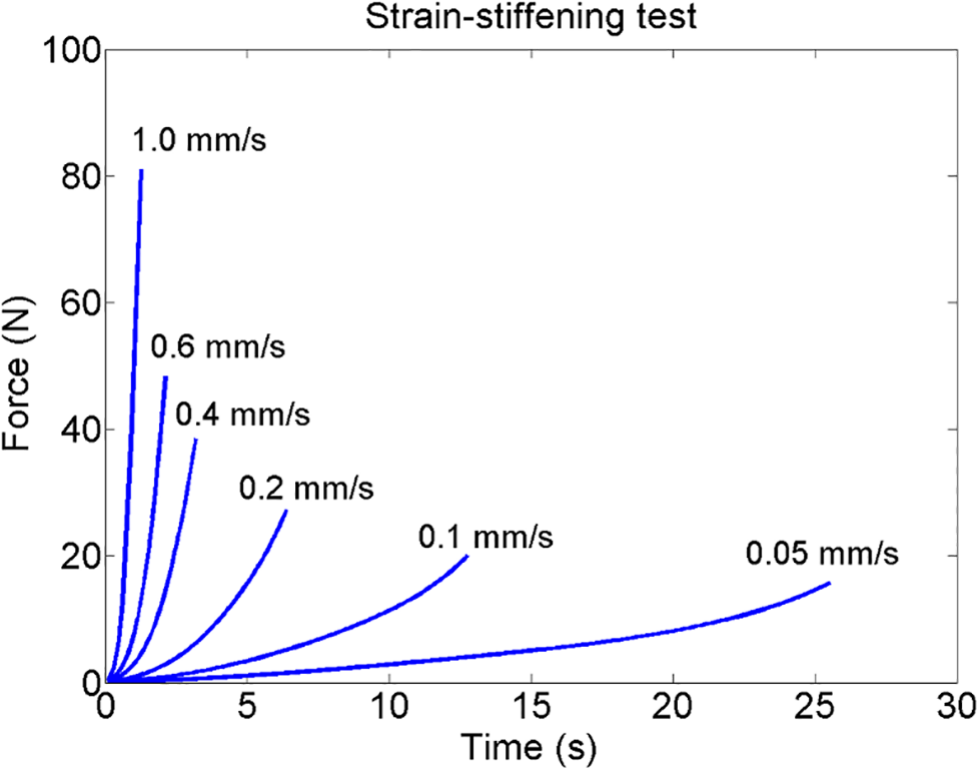
Strain-stiffening test. The strain-stiffening behaviour captured by the poroviscoelastic model where the Achilles tendons subjected to higher strain-rates exhibit a stiffer and more brittle behaviour than when subjected to slower strain-rates.

**Fig 7:**
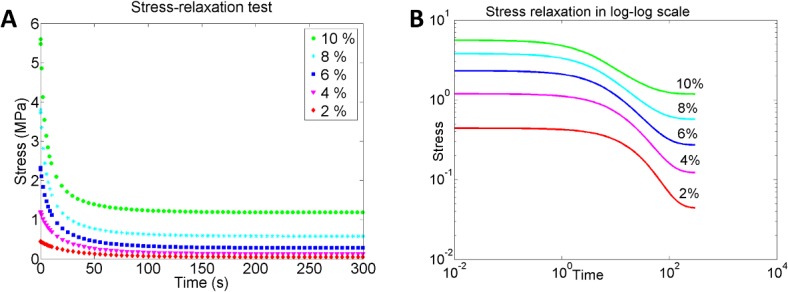
Stress-relaxation test. Stress-relaxation response of the Achilles tendon, as predicted by the material model. Higher strains result in reduced relaxation compared to lower strain magnitudes (A), and demonstrate a slower relaxation rate, as illustrated in the log-log plot (B).

**Fig 8:**
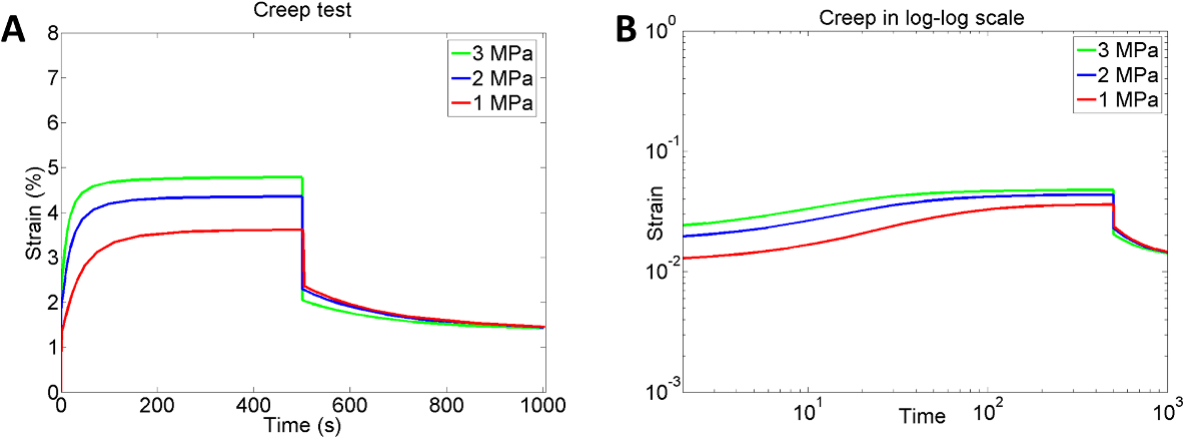
Creep test. A) Model prediction of the creep behaviour in Achilles tendons when subjected to different stress magnitudes. B) The log-log plot shows almost no stress-dependent creep rate behaviour.

Furthermore, our model predicted the characteristic strain-stiffening behaviour of the Achilles tendons. The simulations demonstrated how increased strain-rate leads to a stiffer and more brittle biomechanical response compared to more compliant tendon behaviour during lower strain-rates (Fig 6). The stress-relaxation test on the average tendon showed that the model can predict strain-dependent stress-relaxation behaviour. The tendons subjected to higher strains did not fully recover during the simulated time (300 s) and seem to reach an equilibrium stress. This was in contrast to tendons subjected to lower strains in the relaxation rate (e.g. 2%), which recovered fully after the load was removed (Fig 7A). The model also captures a strain-dependent relaxation rate behaviour where lower level of strains (<6%) results in increased rate of relaxation, whereas higher strains (>6%) lead to slower relaxation (Fig 7B). In the creep test of the average tendon, the model showed the expected viscoelastic creep behaviour where higher levels of stress induces higher creep in the tendon, which returns to an equilibrium strain level when the loading is reduced (Fig 8A). In contrast to the stress-relaxation behaviour, the creep test did not show a load-dependent creep rate or recovery rate in the tendons and predicted only that creep rate is fastest during the first 100 s of loading, independent of the loading magnitude (tested for 1, 2 and 3 MPa, see Fig 8B).

**Fig 9:**
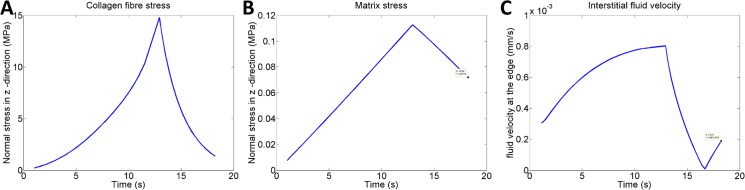
Contribution of tissue constituents. A) The stress in the collagen fibres and B) the stress in the non-fibrillar matrix, during one load cycle. C) Fluid velocities in the tendon during one load cycle (output from highlighted element at the centre edge of the tendon mesh, see Fig 2A). The dip in fluid velocity is an effect of the boundary condition, which creates a propagating wave through the tendon.

The influence of the different tissue constituents (collagen fibres, non-fibrillar matrix and water) on tensile load bearing was investigated by looking at how much each component contributes to fluid velocities and total stress in the tendon (see Fig 9). The results showed that the collagen matrix were the main load-bearing component as the non-fibrillar matrix only contributed to 1% of the total stresses in the tissue. Fluid velocities were in the magnitude of 1 µm/s. The tensile forces in the tendon lead to a volume increase, which produced a negative pore pressure inside the porous structure and a flux of water into the tendon. This behaviour was also observed when the collagen fibres were orientated horizontally such that they could not bear any tensile loads. Despite a small tensile load applied on the average tendon model, the pore pressures were negative and an influx of water was observed. These simulations showed that the poroelastic description of the tendon material model contributes partly to the stress-relaxation behaviour of the tissue but that the equilibrium stress is not due to fluid exudation out of the tissue, but rather due to a decreased pore pressure.

## Discussion

In this study, we have proposed a new material model for tendon that can capture the biomechanical behaviour of the rat Achilles tendon and the role of its structural components during tensile loading. To the best of our knowledge, this is the first poroviscoelastic structural model developed for tendons. The model has been calibrated to experimental data with high accuracy. Moreover, simulations of other loading conditions than those of the experiment (such as strain-rate, stress-relaxation and creep) show that the model can accurately capture the general biomechanical phenomena often reported in tendon experiments.

When comparing with experimental observations, there are some interesting aspects of the predicted biomechanical behaviour of the rat Achilles tendons in this study that need further attention. For example, the stress-relaxation test showed increased rate of relaxation for low strain levels compared to higher strains. Although similar magnitudes of strain (6% as reported in this study) have been reported for alternate stress-relaxation rates [39, 40], literature data is conflicted about the strain-dependent relationship. Some studies have reported that an inverse relationship between strain magnitude and relaxation rate applies for ligaments [41, 42] and that relaxation rates are slower under reduced strains in tendons [41, 43, 44]. However, others have reported that the temporal stress-relaxation is independent of strain values in human cruciate ligaments and human patellar tendons [39]. The discrepancies in results could be due to the origins of the tendons used in the studies as tendons of various anatomical sites have different functions and thus different biomechanical behaviour. Another factor could be age related, as young and still growing tendons have different biomechanical responses than mature tendons to the same loading conditions [45–47]. Moreover, adaptation to load may also play a role during growth. The experimental data used in this study comes from immature rat Achilles tendons that were still growing and had new collagen matrix and limited amounts of cross-links [32]. Thus perhaps future experiments on mature rat Achilles tendons could help elucidate whether these discrepancies in stress-relaxation depend on the age and cross-links in the tendons.

When creep was tested, the model showed almost no strain-dependent recovery rate. Reduced recovery rates compared to relaxation rates have previously also been reported in experiments of for example digital flexor tendons [40]. However, our model assumes aligned collagen fibres as an initial structural condition and does not model the fibre-recruitment process. Thus, it cannot capture the full extent of creep in Achilles tendons under tensile loading. Our optimisation simulations of later loading cycles (cycle 10–12) show a slightly better model fit to experimental data than the early loading cycles (cycle 1–3). This suggests that the model in its current form is somewhat better at representing preconditioned Achilles tendons and that including fibre-recruitment in the future could enable us to better represent creep behaviour in Achilles tendons as well as the load-dependent fibre-orientation process. Fibre-recruitment in intact soft tissues has previously been modelled by viscoelastic or hyperelastic models, where the fibre configuration follows a directional tensor in the continuum description [23, 48] or according to a statistical distribution function [49, 50]. Experimental literature shows that loaded collagen fibres are primarily horizontally aligned but that there could be local fibre dispersion that influences the overall biomechanical response [51, 52]. The tendon model developed in this study has the capacity to initialise with realistic local fibre dispersions that could further improve the results obtained in this study.

The simulations were able to capture the strain-stiffening response of tendons when subjected to increased loading rates. This phenomena has been reported in animal experiments [53] and also in the patellar tendon of clinical patients, where increased loading rates lead to a biomechanical behaviour that corresponded to stiffer tendons and higher Young’s modulus [54]. In fact our model predicts Young’s modulus in the order of 300 MPa and 900 MPa for strain-rates of 0.1 mm/s and 1 mm/s respectively which are similar magnitudes to those reported in experiments (in both our study and others, modulus is measured in the linear region of the mechanical test data) [32, 55].

The simulations in this study showed clearly that the viscoelastic behaviour of the collagen fibres in the rat Achilles tendons were responsible for the main biomechanical response during tensile loading. This agrees with experimental findings that have reported a much higher tissue elastic modulus in tendons along the fibre-aligned direction compared to the transverse [56] and shear directions [57], indicating the structural and load-bearing role of the collagen fibres compared to the non-fibrillar matrix. The five model parameters regulating the viscoelastic behaviour of the collagen fibres (i.e. *E*_1_, *E*_2_, *k*_1_, *k*_2_ and *η*) were more robustly optimised as the collagen fibres were the main load-bearing components (non-fibrillar matrix carried only 1% of the load) and were responsible for the main tissue behaviour. The remaining model parameters belonging to the poroelastic and hyperelastic description of the water and the non-fibrillar matrix, respectively, were also optimised but did not change much from the initial estimations. This is due to the loading scenario to which the model was calibrated. Other alternative loadings, such as shear and transverse stretch are likely to influence the optimisation of the non-fibrillar and fluid parameters more. However, these are very challenging to carry out experimentally. Another remark is that the Matlab optimisation scheme implemented in this study (fminsearch) is sensitive to initial inputs and can potentially get stuck in local minima [58]. This was controlled by manual adjustment of the initial model parameters when necessary. Hence, other non-linear and constrained optimisations schemes should be tested in the future to ensure a robust search for the global optimum.

In general, tensile loading of the tendon demonstrated a volume increase and a decrease in pore pressure, which resulted in positive fluid flux into the tendon. This feature has also been observed in other poroelastic models of soft tissues [59] but is contrary to experimental data where fluid exudation is reported upon tensile loading [60, 61]. In a recent technical study on ligaments, Adeeb, Ali [59] demonstrated that fluid exudation can be achieved by creating barrel shaped geometries (either through initial curvature or by osmotic swelling) or using orthotropic materials with high Poisson’s ratios. Orthotropic material description for rat tail tendon was adopted by Lavagnino, Arnoczky [27] who successfully showed fluid exudation with tensile loading (they predicted same order of magnitude in fluid velocity as those captured by our model). However, their orthotropic material parameters were not fitted to experimental data. Similar limitations apply to our model where the tendon experiments used to optimise the constitutive model expose the tendons to a high stretch. This tension primarily activates the collagen fibres, thus the material properties that affect the volumetric change in the solid part of the poroviscoelastic model are not robustly optimised. Other experimental data and a hyperelastic orthotropic material model for the non-fibrillar solid should be developed in the future to capture the fluid exudation from the tendon.

The non-fibrillar matrix showed viscoelastic behaviour due to the biphasic description and demonstrated fluid flow in the matrix (simulations with horizontally aligned collagen fibres). In tendons, the non-fibrillar matrix is believed to play an essential role in tendon micro-mechanics (although not in the overall tendon response) as the proteoglycan links are responsible for the sliding that occurs between the collagen fibrils when the tissue is stretched [62, 63]. Knowing also that tenocytes and tenoblasts embedded within this matrix are responsive to fluid flow stimulation and strain, and regulate tissue synthesis; our findings indicate that the role of permeability in the proteoglycan matrix, such as transversely isotropic permeability, and the non-fibrillar matrix’s response to loading needs further research in order to capture a more complete picture of how the different structural components of the Achilles tendon are synergised to maintain and restore the tissue’s load-bearing capacity. The findings in this study suggest that the permeability of the tissue may not be as important for the biomechanical behaviour of tendons as it is for the mechanobiological response of the tissue to loading. Henceforth, further experimental data is needed to calibrate permeability parameters of the proposed model for its application on mechanobiological models of tendon repair and homeostasis.

In this study, specimen-specific FE models were developed for each of the experimental samples with unique loading protocol recorded from the experiments. The large variability in the experiments was captured with good accuracy by the results of the tendon simulations. By using specimen-specific models we can capture biological variability instead of only using an average tendon model that may not be representative to an animal population. Variability in our models also enables quantitative analysis of our results and the application of statistical tools on computational results when comparing different simulation groups. In this manner, not only do we get quantitative experimental data but also quantitative computational output, such as constitutive constants and fibre configurations that contain information about the mechanical behaviour of the system. Cook and Purdam [64] have highlighted that applying one treatment to all tendinopathies is not the best clinical approach, but in order to solve this problem more tendon-specific knowledge is required where considering biological variability in the tissues’ structure and composition is essential for an in-depth knowledge about its response to mechanical loading.

In summary, we have presented a novel multi-structural biomechanical model for tendon that can consider the role which the different tissue components play in tensile load bearing. The model presented is able to capture the biomechanical behaviour of rat Achilles tendons under tensile loading with good accuracy, when fitted to specific experimental data. It is able to predict general tendon biomechanical behaviour observed in other experiments, such as stress-relaxation, strain-stiffening and to some extent creep. Our simulations indicate that collagen fibres are the main load-bearing component in rat Achilles tendons under tensile loading, and that stress-relaxation rate in these tendons is inversely correlated to level of strain. This multi-structural model has allowed us to deconstruct and test the different components of the tissue in order to better understand how they interact to create the overall biomechanical response of the tendon.
